# Rheological and volumetric properties of TiO_2_-ethylene glycol nanofluids

**DOI:** 10.1186/1556-276X-8-286

**Published:** 2013-06-13

**Authors:** David Cabaleiro, María J Pastoriza-Gallego, Carlos Gracia-Fernández, Manuel M Piñeiro, Luis Lugo

**Affiliations:** 1 Departamento de Física Aplicada, Facultade de Ciencias, Universidade de Vigo, Vigo 36310, Spain

**Keywords:** Nanofluid, TiO_2_/EG, Anatase, Rutile, Non-Newtonian, Nanoparticles, Density, Thermal expansivity coefficient

## Abstract

Homogeneous stable suspensions obtained by dispersing dry TiO_2_ nanoparticles in pure ethylene glycol were prepared and studied. Two types of nanocrystalline structure were analyzed, namely anatase and rutile phases, which have been characterized by scanning electron microscopy. The rheological behavior was determined for both nanofluids at nanoparticle mass concentrations up to 25%, including flow curves and frequency-dependent storage and loss moduli, using a cone-plate rotational rheometer. The effect of temperature over these flow curve tests at the highest concentration was also analyzed from 283.15 to 323.15 K. Furthermore, the influence of temperature, pressure, nanocrystalline structure, and concentration on the volumetric properties, including densities and isobaric thermal expansivities, were also analyzed.

## Review

### Background

Nowadays, it is well known that the novel proposal of nanofluids represents a valuable way for the development of the heat transfer fluids currently available. Thus, nanofluids have recently emerged with new potential applications in heat exchangers or cooling devices, being widely used in many engineering applications as electronics cooling, vehicle engines, nuclear reactors, energy efficiency enhancers, food industry, air conditioning, refrigeration, and biomedicine [1-4]. As an example, it has been shown that by using nanofluids in radiators, pumps, or compressors in cars, the aerodynamic charge could be reduced, producing fuel savings up to 6% [5]. Therefore, with the aim to improve the heat transfer properties of nanofluids, a considerable amount of research efforts are being devoted to the analysis of their thermal conductivity and convective heat transfer properties. Although it is possible to tailor nanofluids exhibiting negative thermal conductivity enhancement, or a decrease in the effective thermal conductivity of the dispersion if compared with that of the base liquid [6], in most cases, nanofluids exhibit a significant enhancement in thermal conductivity. Therefore, nanofluids are expected to provide optimized convective heat transfer coefficients. However, this type of nanocolloidal dispersion affects also other thermophysical properties than thermal conductivity. Concerning the concentration dependence of nanofluids, a revision of the literature shows, besides the increase in thermal conductivity, decreases of heat capacity and a noticeable increase of density and viscosity, including the possibility of a non-Newtonian behavior. All these properties affect significantly the convective heat transfer coefficient. In addition, as the relation between this coefficient and the involved thermophysical properties could not follow classical laws, it is essentially required to determine accurately their trend with concentration, temperature, and/or pressure.

Recently, Huminic and Huminic [2] have reported a review on the application of nanofluids in various types of heat exchangers as plate, shell and tube, compact, and double pipe heat exchangers. The authors concluded that both the thermophysical properties and type of flow inside the heat exchanger played important roles in the efficiency of the nanofluid as a coolant. Moreover, in most practical applications, the heat transfer fluid is not stationary [3], and consequently, the analysis of the rheological properties is also essential to appropriately determine the increments on the average heat transfer coefficient of the flowing system, which generally increases with the concentration of nanoparticles as well as with the Reynolds number [2]. Numerical results [7] indicate that high-concentration nanofluids of TiO_2_ or Al_2_O_3_ in water exhibit higher heat transfer enhancements and also higher pressure drops. On the other hand, Peyghambarzadeh et al. [4] have experimentally demonstrated, using water- and ethylene glycol (EG)-based nanofluids as cooling agents inside flat aluminum tubes of a car radiator, that the heat transfer behaviors of the nanofluids were highly dependent on particle concentration and flow conditions and otherwise weakly temperature dependent. From the results of Huminic and Huminic [2], it can be concluded that homogeneously dispersed and stabilized nanoparticles enhance the forced convective heat transfer coefficient of the base fluid in a range of 3% to 49%, observing a greater increase with increasing temperature and nanoparticle concentration. Therefore, a proper balance between the heat transfer enhancement and the pressure drop penalty, together with viscosity behavior, should be taken into account when seeking an appropriate nanofluid for a given application.

In addition to the knowledge of the cited rheological behavior, the volumetric properties including the isobaric thermal expansivity coefficient play as well an important role in many heat removal systems involving natural convection. The thermal expansivity coefficient is needed to apply nanofluids in engineering-scale systems [8,9], and this property is usually negligible for metallic oxide particles if compared to that of the base fluids as EG or water. Hence, it is often presumed that this coefficient should decrease with rising concentration of nanoparticles as we have previously reported [10]. Nevertheless, some works [8,9] have found the opposite behavior of the one resulting from considering the fluids to behave separately in the mixture for the case of water-based Al_2_O_3_ nanofluids. This is one of the singular properties of nanofluids that would find a remarkable application in many heat extraction systems using natural convection as a heat removal method [11]. Therefore, more attention should be paid to this magnitude with the goal to understand the complex interaction of nanoparticles with the base fluid molecules, and it could be also a powerful additional tool to characterize nanofluids.

In this work, we focus our attention on the volumetric and rheological behaviors of the suspension of two nanocrystalline forms of TiO_2_ nanoparticles, anatase and rutile, dispersed in pure EG as the base fluid. The influence of the nanocrystalline phase, temperature, pressure, and concentration on the isobaric thermal expansivity coefficient is also analyzed, looking for a verification of the surprising results for different nanofluids found by Nayak et al. [8,9]. In addition to the reasons cited, the selection here of TiO_2_/EG nanofluids is inspired also on several other arguments. First, EG can be used over a wide temperature range. Then, an enhancement in the overall heat transfer coefficient of up to 35% in a compact reactor-heat exchanger, with a limited penalty of increase in pressure drop due to the introduction of nanoparticles, has been reported for TiO_2_/EG nanofluids [3]. Moreover, TiO_2_ is a safe and harmless material for human and animals if compared with other nanomaterials [12]. From a production perspective, these nanoparticles are easily obtained because they are readily produced in large industrial scales. Concerning their physicochemical profile, they have an excellent stability when dispersed in a fluid even without stabilizer addition, and metal oxide nanoparticles are chemically more stable than their metallic counterparts [13]. Finally, remarkably few works are found in the literature [3,14,15] devoted to the study of thermal or rheological properties of TiO_2_/EG nanofluids, and up to our knowledge, their volumetric and viscoelastic properties have never been reported.

The experimental density of stable and homogeneous TiO_2_/EG nanofluids at percent mass concentrations (wt.%) of 1.00, 1.75, 2.50, 3.25, and 5.00, which correspond in percent volume (vol.%), respectively, of 0.29, 0.51, 0.74, 1.04, and 1.51 for anatase and 0.26, 0.47, 0.67, 0.94, and 1.36 for rutile, in wide pressure (from 0.1 to 45 MPa) and temperature (from 283.15 to 343.15 K) ranges was analyzed. From these density data for anatase titanium dioxide-EG nanofluids (A-TiO_2_/EG, from now on, for the sake of brevity) and rutile titanium dioxide-EG nanofluids (*viz.* R-TiO_2_/EG) [16], the derived thermal expansion and thermal compressibility coefficients were studied. Moreover, we have carried out a rheological study on samples of A-TiO_2_/EG and R-TiO_2_/EG nanofluids at mass concentrations of 5.00, 10.00, 15.00, 20.00, and 25.00 wt.%, which correspond to 1.51, 3.13, 4.88, 6.77, and 8.83 vol.% for A-TiO_2_/EG and to 1.36, 2.83, 4.43, 6.16, and 8.08 vol.% for R-TiO_2_/EG, respectively. The effect of the structure of nanoparticles, rutile and anatase, on linear and non-linear tests was analyzed on these samples, and the influence of the temperature was carried out over a temperature range of 283.15 to 333.15 K for the 25 wt.% concentration in both structures.

Several works in the literature have focused on water- or water + EG-based TiO_2_ nanofluids [13,17-24]. Bobbo et al. [17] and Penkavova et al. [18] studied the viscosity of TiO_2_/water nanofluids observing a Newtonian behavior for all compositions, while He et al. [13] concluded that aqueous TiO_2_ nanofluids, with anatase phase and a small amount of rutile phase, show a shear thinning behavior where the shear viscosity tends to be constant at shear rates above 100 s^−1^ and also that the pressure drop of these nanofluids is very close to that of the base liquid. Nevertheless, Tseng and Lin [24] have investigated the rheological behavior of suspensions of anatase TiO_2_ nanoparticles in water (0.05 to 0.12 vol.%), reporting a pseudoplastic flow for most of the shear rates examined, from 10 to 1,000 s^−1^. Moreover, their tests suggest a time-dependent phenomenon, attributing to these suspensions a thixotropic response [24]. Several authors [19-23] have studied thermal conductivity enhancements, higher than 20% [21], increasing the nanoparticle concentration. Concerning volumetric studies in TiO_2_/water nanofluids, only the work by Setia et al. [20] can be cited, where the specific volume for several concentrations up to 0.75 vol.% of TiO_2_ nanoparticles for several temperatures is reported, finding significant deviations from the additive rule [25] for the samples with volume fractions higher than 0.5 vol.%.

Nevertheless, as pointed out above, few studies were focused on the thermophysical or rheological behavior of TiO_2_/EG nanofluids [3,14,15]. Fan et al. [3] determined the thermal conductivity at 303 K for the concentrations 0.5, 2.0, and 4.0 wt.% (corresponding respectively to 0.10, 0.43, and 0.86 vol.%) for TiO_2_/EG nanofluids and their corresponding viscosity in the shear rate range of 1 to 3,000 s^−1^, confirming a Newtonian behavior and the expected increase of viscosity with nanoparticle concentration. Chen et al. [14] have also found a Newtonian behavior for TiO_2_/EG nanofluids containing 0.5, 1.0, 2.0, 4.0, and 8.0 wt.% spherical nanoparticles at 293.15 to 333.15 K and a relative viscosity dependent on particle concentration in a non-linear manner without temperature dependence. On the other hand, Lee et al. [15] have determined temperature-independent thermal conductivity enhancements up to 16% for 5.5 vol.% TiO_2_/EG nanofluids constituted by nanoparticles with rutile and anatase phases. On the other hand, to our knowledge, no evidence on non-Newtonian behavior for TiO_2_/EG nanofluids, or studies about their volumetric behavior, including densities, isothermal compressibility, and isobaric thermal expansivity coefficients, have been reported so far in the literature. Hence, there is a key need to address this issue.

## Methods

**Figure 1:**
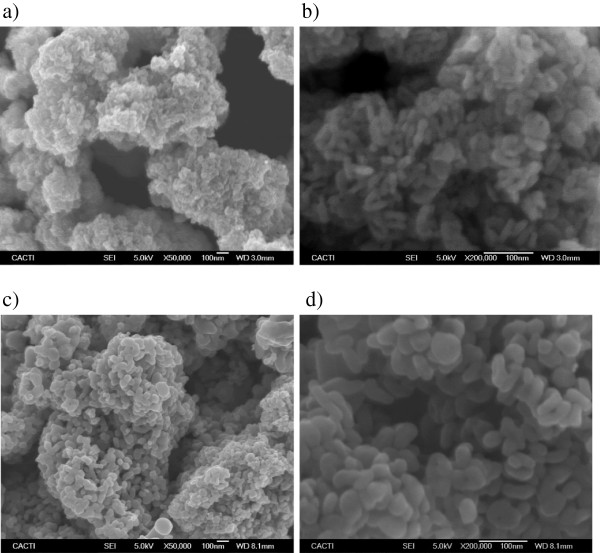
**SEM images of dry A-TiO**_**2 **_**and R-TiO**_**2 **_**nanoparticles.** SEM images of anatase nanoparticles at two magnifications: ×50,000 **(a)** and ×200,000 **(b)**, and rutile nanoparticles at two magnifications: ×50,000 **(c)** and ×200,000 **(d)**.

**Figure 2:**
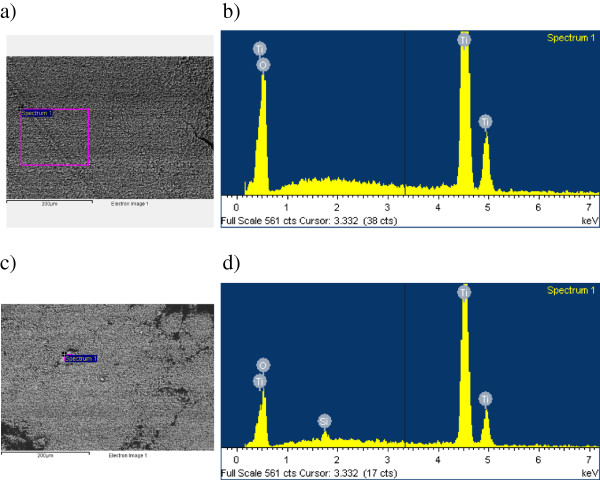
**EDS images and microanalysis of TiO**_**2 **_**nanoparticles.** EDS images of A-TiO_2_**(a)** and R-TiO_2_**(c)** nanoparticles, and microanalysis from the area within the rectangle shown in EDS images for A-TiO_2_**(b)** and R-TiO_2_**(d)** nanoparticles.

Homogeneous and stable suspensions were prepared by dispersing dry TiO_2_ nanoparticles in pure EG. Two types of TiO_2_ powder, corresponding to the pure nanocrystalline anatase and rutile phases, whose descriptions are shown in Table 1, were employed. Although rutile is the stable phase for bulk TiO_2_, the colloidal phase preparation methods for TiO_2_ generally favor the anatase structure [26,27]. Both types of nanoparticles were supplied by SkySpring Nanomaterials, Inc. (Houston, TX, USA) with a reported average size of 10 to 30 nm for rutile and 10 to 25 nm for anatase, with a chemical purity of 99.5% for both cases, while ethylene glycol with a mass purity of 99.5% was supplied by Sigma-Aldrich (St. Louis, MO, USA). With the aim to characterize the morphology of these nanomaterials, both types of TiO_2_ nanoparticles were characterized using the scanning electron microscopy (SEM) technique, obtaining the images with a JEOL JSM-6700 F field emission gun-SEM (Akishima-shi, Japan) operating at an acceleration voltage of 20 kV in a backscattering electron image (yttrium aluminum garnet-type detector). This device incorporates an energy-dispersive X-ray (EDS) spectrometer that was used to chemically characterize the samples. SEM samples were prepared by the deposition of a drop of nanopowder dispersed in methanol (analytical grade) on top of a silica film, dried under atmospheric conditions. SEM images for both types of nanocrystalline structures are shown in Figure 1. The magnification of close agglomerates in micrometers (Figure 1b,d) allows identifying the individual nanoscale globular or nearly spherical particles for anatase and rutile. Average particle sizes were estimated using SEM micrographs by counting a minimum of 100 particles, obtaining values of 35 ± 17 nm for anatase and 47 ± 18 nm for rutile. Figure 2 shows the chemical composition of the samples, obtained from the EDS spectra, determined from the area displayed in Figure 2a,c and represented in Figure 2b,d. The analysis of anatase nanoparticles shows that only Ti and O elements are detectable (Figure 2b), while for rutile, an amount inferior to 1% by mass of Si is present, as shown in Figure 2d, probably due to the silica support. No relevant amounts of other compounds were identified for the samples studied.

**Table 1:** Material description

**Material**	**Supplier**	**Mass purity (%)**	**Medium size (nm)**	**Crystalline structure**	
Anatase titanium dioxide (A-TiO_2_)	SkySpring Nanomaterials	99.5	35 ± 17	Tetragonal	
Rutile titanium dioxide (R-TiO_2_)	SkySpring Nanomaterials	99.5	47 ± 18	Tetragonal	

The preparation of the nanofluid was carried out using the two-step method at the mass concentrations of 1.00, 1.75, 2.50, 3.25, and 5.00 wt.% for volumetric measurements, whereas 5.00, 10.00, 15.00, 20.00, and 25.00 in wt.% concentrations were used for rheological tests, without adding any surfactant, in order to study the effect of nanoparticle aggregation. The uncertainty in the mass compositions for the different studied nanofluids ranges from 0.003% to 0.02%, increasing with the mass concentration. Subsequently, the nanofluids were dispersed by ultrasonic homogenization using a Bandelin Sonoplus HD 2200 (Bandelin Electronic, Berlin, Germany) for 16 min to prevent aggregation. More details about sonication methods have been previously published [28].

Concerning the characterization of the volumetric behavior of the cited R-TiO_2_/EG and A-TiO_2_/EG nanofluids, density measurements were experimentally carried out at concentrations up to 5% in mass fraction from atmospheric pressure up to 45 MPa and from 278.15 to 363.15 K. Temperature and pressure were measured within uncertainties of 0.02 MPa and 0.02 K for pressure and temperature, respectively. Density data were obtained from the period values measured using a commercially available vibrating tube densimeter (Anton Paar DMA 512P, Graz, Austria) with an estimated uncertainty of 5 × 10^−4^ g cm^−3^[29] over the whole pressure and temperature range. More details about the procedure, calibration, temperature, and pressure control can be found in our previous works [10,30,31].

Rheological properties of R-TiO_2_/EG and A-TiO_2_/EG nanofluids were determined using a rotational Physica MCR 101 rheometer (Anton Paar, Graz, Austria), equipped with a cone-plate geometry with a cone diameter of 25 mm and a cone angle of 1°. The cone went down to an imposed gap of 0.048 mm from the plate and covered the whole sample for all tests. The measurement consists of imposing the shear stress to the sample and recording the related shear rate. Temperature is controlled with a Peltier P-PTD 200 (Anton Paar, Graz, Austria), placed at the lower plate, with a diameter of 56 mm without groove. The linear and non-linear tests were developed from torques of 0.1 μNm in the temperature range of 283.15 to 323.15 K, each 10 K. A constant amount of 110 μl of sample was considered [32] for the analysis and was placed on the Peltier plate. Non-linear and linear viscoelastic experiments were carried out with the objective to analyze both relatively large deformations and small-amplitude oscillatory shear. Thus, the flow curves of the samples studied and the frequency-dependent storage (*G*’) and loss (*G*”) moduli were determined. More details about the experimental setup and operating conditions can be found in our previous papers [10,32,33].

## Results and discussion

### Volumetric properties

**Figure 3:**
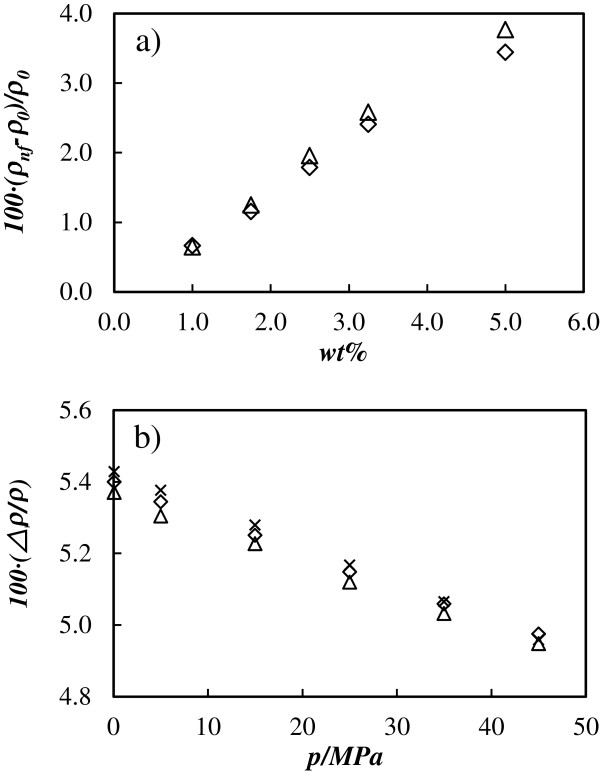
**Average density variations with nanoparticle concentration and pressure. (a)** Enhancement of the density in relation to the base fluid (100 × (*ρ*_nf_*− ρ*_0_)*/ρ*_0_) vs. concentration (wt.%) for both nanocrystalline structures. **(b)** Density diminutions with temperature (−100 × (Δ*ρ*)/*ρ*) vs. pressure (*p*) for the base fluid and both nanocrystalline structures. Cross mark, base fluid; diamond, A-TiO_2_/EG; triangle, R-TiO_2_/EG.

The density values of both sets of nanofluids, A-TiO_2_/EG and R-TiO_2_/EG, at mass fractions up to 5 wt.% were experimentally measured at pressure up to 45 MPa in a wide temperature range of 278.15 to 363.15 K along eight isotherms. Table 2 reports the experimental density data for both nanofluids. The density values range from 1.0627 g cm^−3^ for pure EG, at 0.1 MPa and 363.15 K, up to 1.1800 g cm^−3^ for A-TiO_2_/EG nanofluids and 1.1838 g cm^−3^ for R-TiO_2_/EG nanofluids at 5 wt.%, *p* = 45 MPa, and *T* = 278.15 K. At equivalent temperature, pressure and concentration, the density values of the A-TiO_2_/EG are lower than those of R-TiO_2_/EG, excepting the 1 wt.% sample, for which they agree to within the experimental uncertainty. Density values increase with nanoparticle concentration as expected, as shown in Figure 3a where the increments in relation to the base fluid reference value at different concentrations are shown, with higher increments also for the rutile nanocrystalline structure, reaching values of 3.8%. We have found that these increments with concentration are almost temperature and pressure independent. For a given concentration, density data show pressure and temperature dependences similar to the base fluid, increasing with pressure and decreasing with temperature. The average percentage density increments with pressure range between 1.5% at the lowest temperature and 2% at the highest temperature. On the other hand, the average percentage density diminutions with temperature at different pressures are gathered in Figure 3b, showing decreases between 5% and 5.4%. These temperature variations are very similar for both nanocrystalline structures and the base fluid, as can be appreciated in this figure.

**Table 2:** **Density ( *ρ *), isobaric thermal expansivity (*α***_***p***_**), and isothermal compressibility (*κ***_***T***_**) of A-TiO**_**2**_**/EG and R-TiO**_**2**_**/EG nanofluids**

	***p *** **(MPa)**	***ρ *** **(g·cm**^**−3**^**)**			**10**^**4**^**·** ***α***_***p ***_**(K**^**−1**^**)**			**10**^**4**^**·** ***κ***_***T ***_**(MPa**^**−1**^**)**		
		***T *** **= 283.15 K**	***T *** **= 313.15 K**	***T *** **= 343.15 K**	***T *** **= 283.15 K**	***T *** **= 313.15 K**	***T *** **= 343.15 K**	***T *** **= 283.15 K**	***T *** **= 313.15 K**	***T *** **= 343.15 K**
Base fluid (EG)	0.10	1.1202	1.0989	1.0772	6.31	6.52	6.73			
	1.00	1.1206	1.0993	1.0776	6.30	6.51	6.72	3.52	3.89	4.34
	20.00	1.1279	1.1073	1.0861	6.09	6.27	6.43	3.34	3.69	4.08
	40.00	1.1353	1.1152	1.0950	5.89	6.03	6.14	3.33	3.66	4.05
	45.00	1.1373	1.1174	1.0973	5.84	5.97	6.07			
A-TiO_2_/EG (1.75 wt.%)	0.10	1.1327	1.1117	1.0901	6.20	6.43	6.66			
	1.00	1.1332	1.1121	1.0905	6.20	6.42	6.65	3.35	3.61	3.97
	20.00	1.1407	1.1200	1.0988	6.06	6.23	6.37	3.38	3.63	4.00
	40.00	1.1482	1.1280	1.1076	5.92	6.03	6.09	3.27	3.51	3.85
	45.00	1.1503	1.1300	1.1100	5.89	5.99	6.03			
A-TiO_2_/EG (5.00 wt.%)	0.10	1.1584	1.1366	1.1147	6.42	6.51	6.59			
	1.00	1.1589	1.1370	1.1150	6.41	6.50	6.58	3.61	3.96	4.33
	20.00	1.1667	1.1450	1.1239	6.21	6.29	6.36	3.35	3.65	3.97
	40.00	1.1745	1.1535	1.1324	6.02	6.08	6.15	3.39	3.70	4.02
	45.00	1.1766	1.1558	1.1349	5.97	6.03	6.10			
R-TiO_2_/EG (1.75 wt.%)	0.10	1.1339	1.1126	1.0910	6.15	6.41	6.67			
	1.00	1.1343	1.1129	1.0914	6.14	6.40	6.66	3.62	0.03	4.50
	20.00	1.1414	1.1209	1.1001	5.93	6.16	6.39	3.28	3.61	3.98
	40.00	1.1491	1.1290	1.1093	5.71	5.92	6.12	3.45	3.82	4.24
	45.00	1.1513	1.1314	1.1113	5.65	5.85	6.04			
R-TiO_2_/EG (5.00 wt.%)	0.10	1.1622	1.1405	1.1184	6.24	6.43	6.63			
	1.00	1.1626	1.1409	1.1188	6.23	6.42	6.62	3.52	3.75	4.07
	20.00	1.1706	1.1489	1.1271	6.10	6.26	6.40	3.41	3.63	3.93
	40.00	1.1779	1.1570	1.1362	5.98	6.09	6.18	3.34	3.55	3.83
	45.00	1.1802	1.1592	1.1382	5.95	6.05	6.12			

With the aim to report a generalized temperature and pressure correlation of the volumetric behavior of the measured base fluid and nanofluids, the specific volumes (*v = 1/ρ*), using the following expression [34], were adjusted to the experimental data:

(1) $$v\left(T,p\right)\;=v\left({T,p}_{\mathrm{ref}}\right)\cdot \left(1-\frac{{p-p}_{\mathrm{ref}}}{B\left(T,p\right)}\right),$$

where the reference pressure, *p*_*ref*_, was taken as 0.1 MPa. The dependence of the specific volume at this pressure was described by using the following expression [35,36].

(2) $$\begin{array}{l} v\left({T,p}_{\mathrm{ref}}\right)=v_{\mathrm{ref}}\left(T_{\mathrm{ref}},p_{\mathrm{ref}}\right)\\ \;\;\;{\times e}^{\left({a.\theta +b\cdot \theta }^{2}\right)}\;\mathrm{with}\;\theta ={T-T}_{\mathrm{ref}}, \end{array}$$

where *a*, *b*, and *v*_ref_(*T*_ref_,*p*_ref_) are the adjustable parameters, *v*_ref_(*T*_ref_,*p*_ref_) being the specific volume at the reference temperature *T*_ref_ = 278.15 K and pressure *p*_ref_ = 0.1 MPa. These coefficients, whose values are given in Table 3, were fitted for the base fluid and the different nanofluids with standard deviations lower or equal than 2.8 cm^3^ g^−1^. The bulk modulus *B*(*T*, *p*) was adjusted as a function of pressure and temperature with the following polynomial:

(3) $$\begin{array}{ll} \;B\left(T,p\right) & =B\left(p_{\mathrm{ref}},T_{\mathrm{ref}}\right)+c\cdot \theta +{d\cdot \theta }^{2}+e\cdot \mathrm{\Delta p}\\ & \;+{f\cdot \Delta p}^{2}\;\mathrm{with}\;\mathrm{\Delta p}\\ & ={p-p}_{\mathrm{ref}}\;. \end{array}$$

**Table 3:** **Density correlation coefficients and standard deviations ( *σ *) for the base fluid (EG) and the nanofluids**

	**Base fluid**	**A-TiO**_**2**_**/EG (wt.%)**					**R-TiO**_**2**_**/EG (wt.%)**				
		**1.00**	**1.75**	**2.50**	**3.25**	**5.00**	**1.00**	**1.75**	**2.50**	**3.25**	**5.00**
10^3^·*a* (°C^−1^)	0.62714	0.62327	0.61646	0.62116	0.63558	0.64060	0.61708	0.61084	0.62243	0.62955	0.62042
10^6^·*b* (°C^−2^)	0.35343	0.30347	0.38267	0.25865	0.17013	0.14365	0.38319	0.43431	0.24473	0.23998	0.32687
10^4^·*σ* (cm^3^ g^−1^)	1.1	1.2	1.2	1.9	1.4	2.8	1.6	1.4	1.8	1.3	1.1
*B*(*p*_ref_*,T*_ref_) (MPa)	2,875.23	2,813.30	3,016.52	2,732.87	2,840.25	2,798.17	2,796.391	2,782.86	2,744.918	2,619.262	2,865.778
*−c* (MPa °C^−1^)	9.1949	8.8432	6.1026	7.7217	10.4348	8.8384	9.8265	9.8347	10.4074	8.6823	5.4028
10^2^·*d* (MPa °C^−2^)	0.3779	0.4173	−0.2270	0.5231	2.44	1.61	1.61	1.23	2.45	0.89114	−1.48
*e*	5.123	5.727	−1.559	11.030	7.262	9.430	8.211	13.951	10.066	17.127	3.220
*−*10^3^*·f* (MPa^−1^)	57.3	−12.3	−49	−103.1	−50.9	108.5	50.8	190.2	71.4	187.5	12.3
10^4^·σ* (cm^3^ g^−1^)	0.7	0.8	1.4	0.9	0.9	1.4	0.9	1.0	1.0	1.3	1.2

The values of *B*(*p*_ref_,*T*_ref_), *c*, *d*, *e*, and *f* were determined by fitting Equation 1 to all the experimental data at pressures different than *p*_ref_ by a least squares method using a Marquardt-Levenberg-type algorithm. For the base fluid and all the studied nanofluids, the standard deviations obtained with this correlation are lower than or equal to 1.4 × 10^−4^ cm^3^ g^−1^, and the coefficients are given in Table 3.

**Figure 4:**
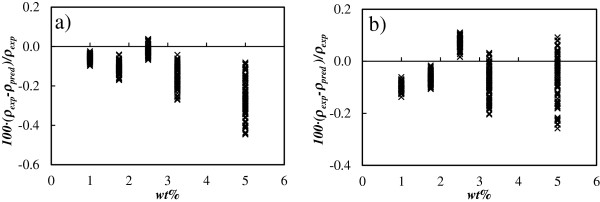
**Percentage deviations between experimental and predicted densities.** Deviations between experimental density data (*ρ*_exp_) and predicted values (*ρ*_pred_) by Equation 4 vs. mass concentration (wt.%) for **(a)** A-TiO_2_/EG and **(b)** R-TiO_2_/EG nanofluids.

Although viscosity, heat capacity, and thermal conductivity are the main parameters involved in the calculation of the heat transfer rate of a nanofluid, the precise determination of density is also relevant because, as commented above, these properties may be quite different from those of the original pure fluid, and it can lead to erroneous mass balances. As we have pointed out, significant variations in density can be achieved when temperature, pressure, concentration, or the type of nanocrystalline structure are analyzed in detail. In order to check some conventional assumptions [3,20], we have determined the ideal nanofluid density from the nanoparticle and base fluid densities according to [25]:

(4) $$\rho_{\mathrm{nf}}=\phi \cdot \rho +\left(1-\varphi \right)\cdot \rho_{0},$$

where ϕ is the volumetric fraction of nanoparticles and the subscripts np, *0*, and nf refer to the nanoparticles, base liquid, and nanofluids, respectively. The densities of anatase and rutile titanium oxide are, respectively, 3.830 and 4.240 g cm^−3^[37]. With the aim to evaluate the goodness of this estimation, our experimental values were compared with those predicted using this equation. It was found that this equation overpredicts the density of the nanofluids studied in this work with deviations that it can reach 0.5% for A-TiO_2_/EG and 0.3% for R-TiO_2_/EG at the highest concentrations and temperatures, as shown in Figure 4.

Isobaric thermal expansivity, *α*_*p*_, and isothermal compressibility, *κ*_*T*_, coefficients can be determined from specific volume correlations using their respective thermodynamic definitions according the following expressions:

(5) $$\alpha_{p}\;=\frac{1}{v}\cdot {\left(\frac{\partial v\left(T,p\right)}{\partial T}\right)}_{p}=\frac{\frac{\partial v\left({T,p}_{\mathrm{ref}}\right)}{\partial T}}{v\left({T,p}_{\mathrm{ref}}\right)}+\frac{{\left(\frac{\partial B\left(T,p\right)}{\partial T}\right)}_{p}\mathrm{\cdot \Delta p}}{B\left(T,p\right)\cdot \left[B\left(T,p\right)-\mathrm{\Delta p}\right]},$$

(6) $$\begin{array}{l} \kappa_{T}\;=-\frac{1}{v}\\ \;\;\cdot {\left(\frac{\partial v\left(T,p\right)}{\partial p}\right)}_{T}=\frac{B\left(T,p\right)-{\left(\frac{\partial B\left(T,p\right)}{\partial p}\right)}_{T}\mathrm{\cdot \Delta p}}{B\left(T,p\right)\cdot \left[B\left(T,p\right)-\mathrm{\Delta p}\right]}. \end{array}$$

In Table 2, the values calculated for *α*_*p*_ and *κ*_*T*_ are reported for some temperatures and pressures for the base fluid (EG) and both nanofluids at two different concentrations (1.75 and 5.00 wt.%). The estimated uncertainties for *α*_*p*_ and *κ*_*T*_ are 4% and 2%, respectively. The *α*_*p*_ values for both the base fluid and R-TiO_2_/EG and A-TiO_2_/EG nanofluids decrease when pressure rises (up to 9.8% for the base fluid) and increase with temperature (up to 6.6% for the base fluid). Concerning the concentration dependence, first, we have found that the *α*_*p*_ values of nanofluids are very similar to or lower than those of EG, achieving decreases up to 1.0% and 1.9% for A-TiO_2_/EG and R-TiO_2_/EG nanofluids, respectively. These results are opposite to those previously found by Nayak et al. [8,9], reporting a significant increase in this property compared to the base fluid for water-based Al_2_O_3_, CuO, SiO_2_, and TiO_2_ nanofluids. It should be mentioned that Nayak et al. have determined the isobaric thermal expansivities by measuring the bulk variation with temperature for the samples in a glass flask with a long calibrate stem. Consequently, further studies about this property are still needed on EG- or water-based nanofluids. On the other hand, the *κ*_*T*_ values of the studied samples do not exhibit evident concentration or nanocrystalline structure dependence (or these differences are within the uncertainty). The *κ*_*T*_ values decrease when the pressure rises and increase with the temperature along the isobars for both the base fluid and nanofluid samples, as can be seen in Table 2.

**Figure 5:**
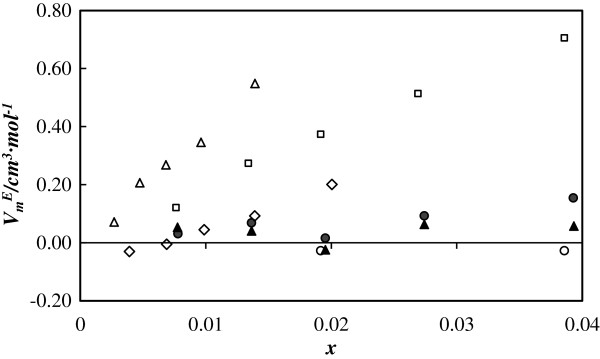
**Excess molar volumes of nanofluids**$V_{m}^{E}$**.** Molar excess volumes vs. molar fraction for different EG nanofluids at 303.15 K and 20 MPa. Filled circle, A-TiO_2_/EG; filled triangle, R-TiO_2_/EG; empty triangle, Fe_3_O_4_/EG [38]; empty diamond, Fe_2_O_3_/EG [38]; empty circle, (48-nm ZnO)/EG [39]; empty square, (4.6-nm ZnO)/EG [39].

In order to compare the volumetric behavior of the nanofluids with the ideal fluid behavior, excess molar volumes, $V_{m}^{E}$, were calculated [10,38]. Figure 5 shows an expansive volumetric behavior for both A-TiO_2_/EG and R-TiO_2_/EG. This behavior has also been found for other pure EG-based nanofluids, and it is contrary to that presented by nanofluids which use water or EG + water as the base fluid [28]. Excess molar volumes for A-TiO_2_/EG increase slightly with nanoparticle concentration ranging from 0.03 up to 0.11 cm^3^ mol^−1^, which correspond to a variation in the molar volume between 3.3% and 14.3%. Concerning R-TiO_2_/EG, its behavior is closer to ideal, and it is almost concentration independent with a maximum variation in volume of 4.6%. No significant temperature or pressure dependences for this property were found.

### Rheological behavior

**Figure 6:**
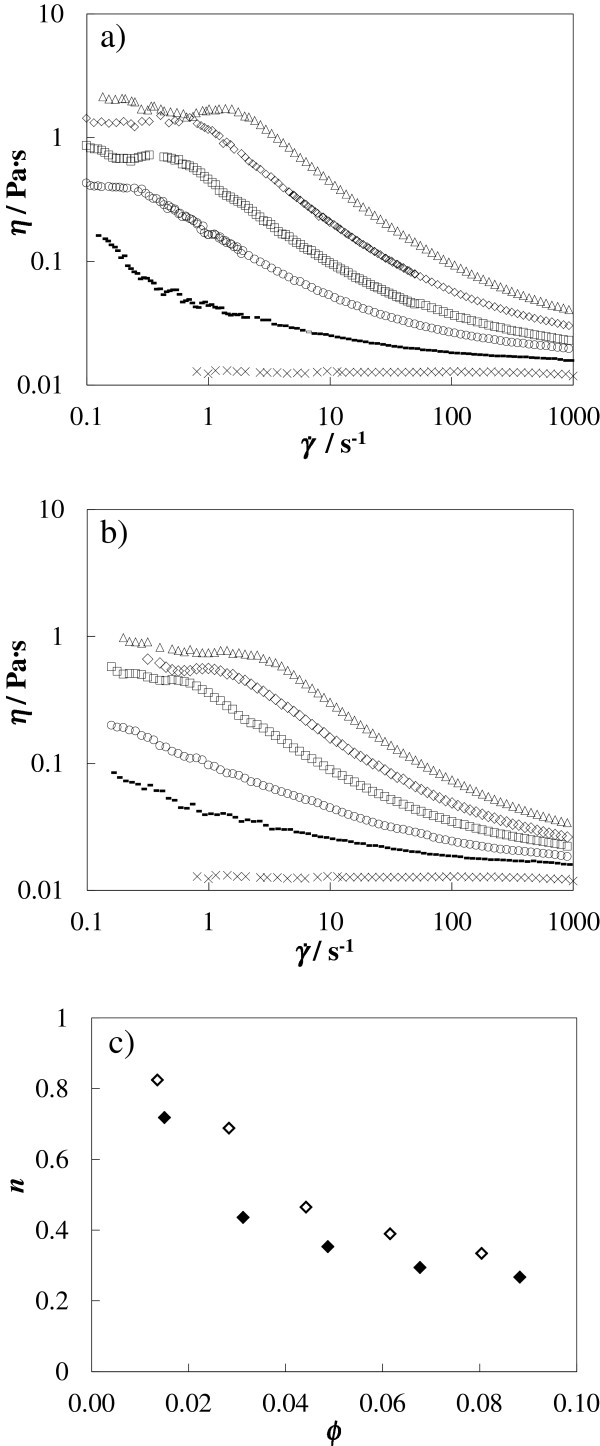
**Viscosity (*η*) vs. shear rate (**$\overset{˙}{\gamma }$**) of EG/TiO**_**2 **_**nanofluids at different concentrations.** Flow curves for **(a)** A-TiO_2_/EG and **(b)** R-TiO_2_/EG at 303.15 K and at different mass concentrations: cross mark, EG; line, 5 wt.%; circle, 10 wt.%; square, 15 wt.%; diamond, 20 wt.%; triangle, 25 wt.%. **(c)** Flow behavior index (*n*) vs. volume fraction (ϕ) for A-TiO_2_/EG (filled diamond) and R-TiO_2_/EG (empty diamond) at 303.15 K.

As pointed out, only a reduced number of studies about the rheological behavior of nanofluids can be found in the literature, and there are inconsistencies such as Newtonian and non-Newtonian behaviors reported for the same nanofluid as well as discrepancies in the effects of temperature, particle size, and shape, and high shear viscosity values [40-44]. In this context, a key issue is to obtain nanofluid structural information, and one of the feasible methods is through detailed rheological analyses [45]. In this work, two types of studies have been carried out. Viscosity as a function of shear rate, the so-called flow curve, was determined for both nanofluids at 303.15 K and at five different mass concentrations (5, 10, 15, 20, and 25 wt.%). The applied torques started from 0.1 μNm, covering shear rate ranges from 0.1 to 1,000 s^−1^. Figure 6a,b shows these flow curves for both nanofluids at different concentrations. Unlike the base fluid, both sets of nanofluids present a clear shear thinning (pseudoplastic) non-Newtonian behavior. In the lowest shear rate region, Newtonian plateaus are easily identified as the concentration rises. This non-Newtonian behavior opposes that reported previously by Chen et al. [14] that studied EG-based nanofluids containing 0.5 to 8.0 wt.% spherical TiO_2_ nanoparticles. Chen et al. [14] affirmed that a Newtonian behavior is found at a shear rate higher than 0.05 s^−1^. It should be taken into account that our viscosity results for Newtonian EG agree with those of Chen et al. [14] within an average deviation of 1.5% [32]. The controversies found in the literature on rheological studies indicate that the specific properties of the nanoparticles such as shape, structure, and size, and the interaction between the base liquid and nanoparticles can play an essential role in determining the rheological behavior of nanofluids. However, in this case, the main reasons of the different rheological behavior on TiO_2_/EG nanofluids may be attributed to the following: (1) the range of nanoparticle concentration studied by Chen et al. [14] (<8 wt.%) is lower than those analyzed in this work (<25 wt.%), (2) the range of shear stress studied in this work covers a wider area, and it is here where shear thinning appears, (3) the minimum shear rate which the equipment can reach is decisive to determine the first Newtonian plateau, especially at low nanoparticle concentration, and finally (4) the different stability and aggregation of particles affect flow conditions because the effective mass concentration can be higher than the actual solid mass.

The Ostwald-de Waele model (Power law) was used to describe the experimental shear dynamic viscosity data, *η*, as a function of the shear rate, γ, in the shear thinning region for each concentration of both sets of nanofluids by using the following expression [46-48]:

(7) $$\eta =\mathrm{K\cdot}{\overset{˙}{\gamma }}^{n-1},$$

where the adjustable parameters *K* and *n* are the flow consistency factor and the flow behavior index, respectively. Good adjustments are obtained for all studied nanofluid samples, reaching percentage deviations in shear dynamic viscosity around 3%. At the same mass concentration, the flow behavior index values for R-TiO_2_/EG nanofluids are higher than those for A-TiO_2_/EG, as shown in Figure 6c. These *n* values range from 0.27 to 0.72 for A-TiO_2_/EG and from 0.33 to 0.83 for R-TiO_2_/EG, decreasing near-exponentially when the volume fraction increases, which evidences that the shear thinning behavior is more noticeable when the nanoparticle concentration increases. The *n* values are similar to those typically obtained for common thermoplastics [49]. It must also be pointed out that although this model offers a simple approximation of the shear thinning behavior, it does not predict the upper or lower Newtonian plateaus [47].

**Figure 7:**
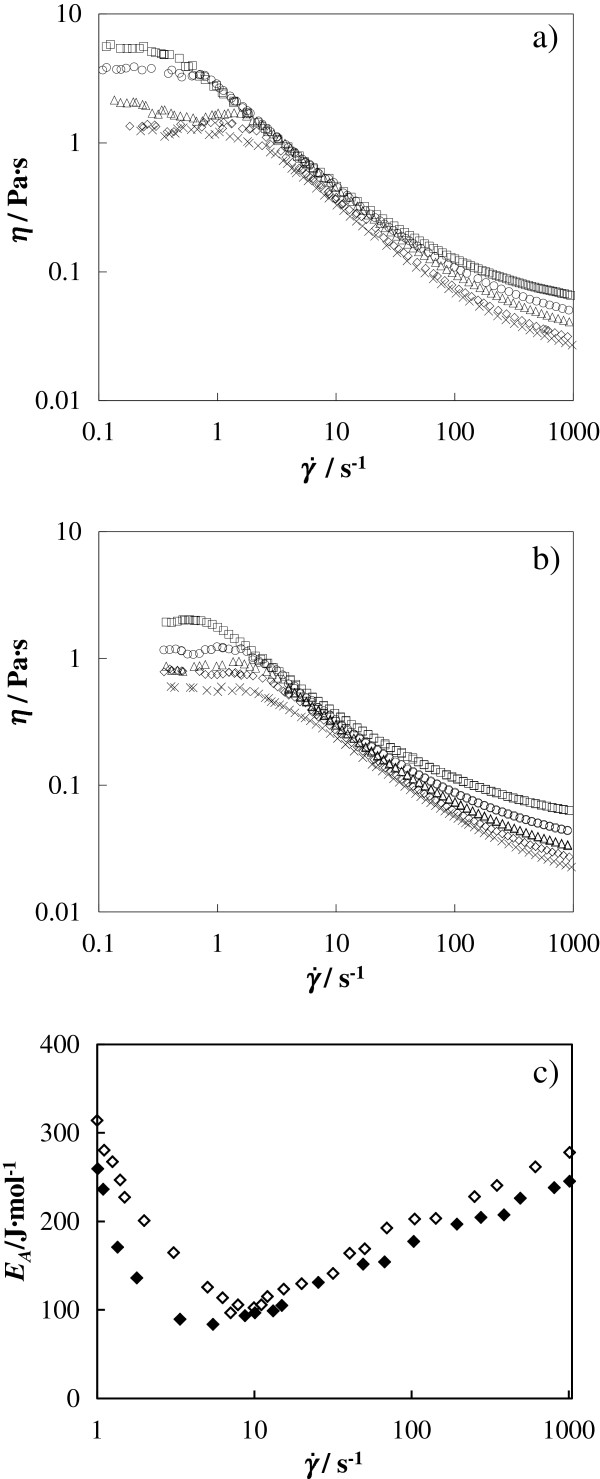
**Viscosity (*η*) vs. shear (**$\overset{˙}{\gamma }$**) rate of EG/TiO**_**2 **_**nanofluids at different temperatures.** Flow curves for **(a)** A-TiO_2_/EG and **(b)** R-TiO_2_/EG at 25 wt.% mass concentration and at different temperatures: square, 283.15 K; circle, 293.15 K; triangle, 303.15 K; diamond, 313.15 K; cross mark, 323.15 K. **(c)** Energy of activation to fluid flow (*E*_*a*_) vs. shear rate for A-TiO_2_/EG (filled diamond) and R-TiO_2_/EG (empty diamond) 25 wt.% nanofluids.

As a further test, the influence of temperature on the flow curves was studied for the highest mass concentration (25 wt.%) for both nanofluids between 283.15 and 323.15 K, as shown in Figure 7a,b, respectively. In these flow curves, we can observe the diminution of viscosity when the temperature rises, as Chen et al [14] had found in their study between 293.15 and 333.15 K. Nevertheless, the shear viscosities reported in this work show a temperature dependence very influenced by the shear rate value. Moreover, we can observe that the shear viscosity is nearly independent of temperature at a shear rate around 10 s^−1^ for both A-TiO_2_/EG and R-TiO_2_/EG nanofluids, which is not the case at a high or low shear rate. On the other hand, at the same concentration and temperature, A-TiO_2_/EG nanofluids present higher shear viscosities than R-TiO_2_/EG nanofluids for all shear rates. These viscosity differences increase with concentration. Applying the Ostwald-de Waele model on these flow curves at different temperatures, we have also obtained good results, finding that *n* values increase with temperature. This may be a result of the temperature effect on the better nanoparticle dispersion. Similar increases of the flow behavior index were also determined previously [50,51].

The influence of temperature, *T*, on the viscosity at each shear rate can be expressed in terms of an Arrhenius-type equation [52,53]:

(8) $$\eta ={\mathrm{A\cdot}e}^{E_{a}/\left(\mathrm{R\cdot T}\right)},$$

where *R* is the universal gas constant and *A* and *E*_*a*_ are the fitting parameters of the pre-exponential factor and energy of activation to fluid flow, respectively. This equation describes adequately the temperature dependence of the shear viscosity of the studied nanofluids. Figure 7c shows the obtained *E*_*a*_ values vs. shear rate for the 25 wt.% concentration of A-TiO_2_/EG and R-TiO_2_/EG nanofluids. It is generally accepted that higher *E*_*a*_ values indicate a faster change in viscosity with temperature and high temperature dependency of viscosity [50]. Thus, lower *E*_*a*_ values found for A-TiO_2_/EG indicate an inferior temperature influence on viscosity for this nanofluid. Moreover, at shear rates around 6 s^−1^ for A-TiO_2_/EG and around 8 s^−1^ for R-TiO_2_/EG, a minimum of the energy of activation was detected, as can be observed in Figure 7c. The values obtained here for A-TiO_2_/EG and R-TiO_2_/EG are similar to those obtained by Abdelhalim et al. [54] for gold nanoparticles in an aqueous solution.

**Figure 8:**
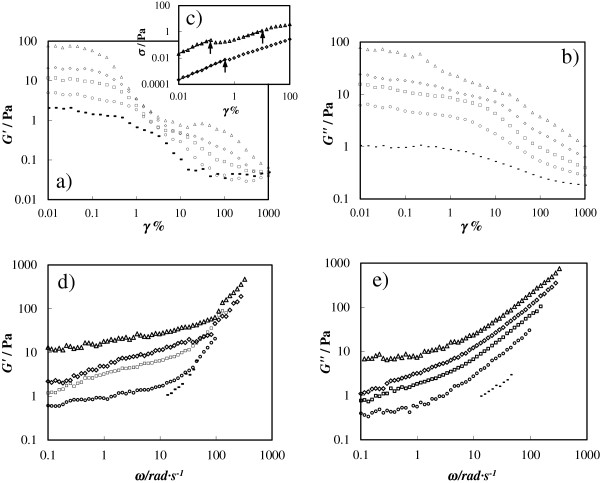
**Storage (*G*’) and loss (*G*”) moduli. (a)** Storage modulus, **(b)** loss modulus, and **(c)** shear stress (*σ*) as a function of strain (*γ*) at an angular frequency of 10 rad s^−1^ and a temperature of 303.15 K for different concentrations of A-TiO_2_/EG. **(d)** Storage and **(e)** loss moduli as a function of frequency (*ω*) at a strain of 0.1% and a temperature of 303.15 K for different concentrations of A-TiO_2_/EG. Line, 5 wt.%; circle, 10 wt.%; square, 15 wt.%; diamond, 20 wt.%; triangle, 25 wt.%.

In addition, linear viscoelastic oscillatory experiments were performed for A-TiO_2_/EG in order to study their mechanical properties under small-amplitude oscillatory shear. The power of these tests is that stress can be separated into two terms and the elastic or storage modulus can be determined. Then, it can be established whether the nanofluid behaves as the base fluid without agglomerates or alternatively as a solid with a certain level of agglomerates due to the increase in the interactions and collisions among particles that lead to gel formation [55]. First, with the aim to identify the linear viscoelastic region, strain sweep tests (for strains between 0.01% and 1,000%) were carried out at 10 rad s^−1^ (see Figure 8a,b). Smaller strain amplitudes were not considered due to equipment conditions as the strain waveform was not sinusoidal due to the presence of experimental noise. A linear regime was found, over which *G*’ and *G*” remain constant at low strains with critical strains lower than 1%, which are weakly concentration dependent whereas the stress upper limit of the linear viscoelastic regime region increases with concentration. After this critical strain, *G*’ and *G*” decrease as the strain increases in two steps, which may correspond to, first, the break of the structure and then the orientation of agglomerates aligned with the flow field at large deformations [55]. This two-step decrease presents two peaks, which become more evident at higher concentrations, that were previously described in the literature as an attractive gel structure [55,56]. Figure 8c shows the strain dependences of the shear stress for the deformation tests where the strains at these two peaks are identified with arrows. The loss modulus clearly decreases at a strain beyond 1%, and no overshoot trend is observed as found on other nanofluids [32].

Frequency sweep tests (for angular frequencies between 0.1 and 600 rad s^−1^) were performed for A-TiO_2_/EG nanofluids, and the evolution of each modulus with the oscillation frequency was obtained, as shown in Figure 8c,d. These experiments were carried out in the linear viscoelastic region using a constant strain value of 0.1% for all nanofluids. Both moduli increase with concentration at a given constant frequency which means that when the nanoparticle content is increased, the hydrodynamic interactions as well as the probability of collision become important, enhancing the aggregation processes. In all cases, the elastic modulus is higher than the viscous one at low frequencies, while the contrary occurs at high frequencies, where the suspensions behave like a liquid. Crossover frequencies, where *G*’ = *G*” and a change in the viscoelastic behavior is detected, increase with the concentration of nanoparticles from around 4 rad s^−1^ at a concentration of 10 wt.% to 15 rad s^−1^ at 25 wt.%. That is in agreement with the fact that the degree of agglomeration of the particles is more important at the highest concentrations, but the alignment with the flow of the aggregates is achieved in a shorter time for higher concentrations. This analysis was not carried out for the lowest nanofluid concentration (5 wt.%) due to the availability of the minimum torque of the used device. Moreover, it should be taken into account that those data at elevated frequencies in which problems of inertia of equipment appear were not considered. This was done by taking into consideration the relationship between the complex viscosity and the frequency. The loss and storage moduli increase with frequency especially at frequencies higher than 10 rad s^−1^. It can be also observed that the elastic modulus data fall on a straight line for the highest frequencies. Finally, we want to point out that the increase in nanoparticle concentration leads to an increase in the formation of agglomeration of the particle, but even the concentration of 5 wt.% for A-TiO_2_/EG nanofluid does not follow the conventional Cox-Merz rule [57], ${\left|\eta^{*}\left(\omega \right)\right|\approx \eta \left(\overset{˙}{\gamma }\right)]}_{\overset{˙}{\gamma }=\omega }$, η^*^ being the complex viscosity η* ≡ (*G´* + *iG´´*)/*ω*, which is often valid for Newtonian or non-structured fluids. Our data demonstrate the Cox-Merz rule to become more inapplicable as the concentration of nanoparticles increases. Moreover, it is illustrated that the addition of nanoparticles makes the difference |η*| − *η* increase as $\overset{˙}{\gamma },\omega \to 0$ for all A-TiO_2_/EG concentrations. This behavior was previously found by Haleem and Nott [58] for suspensions of rigid spheres in semi-dilute polymer solutions. These authors attributed this anomalous behavior to the fact that an anisotropic particle microstructure can form at steady shear even in the limit $\overset{˙}{\gamma }\to 0$, while it cannot reach it for small-amplitude oscillatory shear. Up to our knowledge, no previous results were published on the Cox-Merz rule of nanofluids, and therefore, more studies exploring other nanofluid types are necessary.

## Conclusions

The density values for R-TiO_2_/EG are higher than those for the A-TiO_2_/EG nanofluid at the same temperature, pressure, and mass concentration. The enhancement of density in relation to the base fluid is also higher for rutile nanofluids, reaching values of 3.8% at the highest concentration. These increments with the concentration are almost temperature and pressure independent. The isobaric thermal expansivity values of A-TiO_2_/EG and R-TiO_2_/EG nanofluids decrease when the pressure rises and increase with temperature as the base fluid does, while we have found that these values for the nanofluids are very similar to or lower than those for the base fluid, achieving decreases up to 2%. The analyzed nanofluids present an expansive volumetric behavior which is more pronounced in A-TiO_2_/EG. This expansive behavior is also found for other EG-based nanofluids. Contrarily to what was previously published, a shear thinning non-Newtonian behavior was found for both sets of TiO_2_/EG nanofluids. As the concentration rises, Newtonian plateaus are found at the lowest shear rate and the shear thinning regions can be described using the Ostwald-de Waele model. At the same temperature and concentration conditions, A-TiO_2_ nanofluids show higher shear dynamic viscosity for all the shear rates. Minima in the energy of activation were found at shear rates around 6 s^−1^ for A-TiO_2_/EG and 8 s^−1^ for R-TiO_2_/EG when the shear dynamic viscosity data were fitted for the 25 wt.% concentrations using an Arrhenius-type equation. Finally, viscoelastic oscillatory experiments were performed for A-TiO_2_. The two-step decrease of the loss modulus present in the deformation tests evidence an attractive gel behavior of the studied nanofluids. Finally, the A-TiO_2_/EG nanofluid does not follow the conventional Cox-Merz rule. The differences between the viscosities determined in steady shear and the dynamic viscosities from the oscillatory rate are higher when the nanoparticle concentration increases.

## Competing interests

The authors declare that they have no competing interests.

## Authors' contributions

DC performed the nanofluid sample characterization and experimental measurements and participated in the redaction and the critical evaluation of experimental results. MJPG contributed with the selection of the optimal experimental setting and type of tests to be performed. CGF participated in the critical evaluation of experimental and theoretical results. MMP analyzed the data and participated in the structuring of the work. LL conceived the study, developed its design, and coordinated the redaction of the manuscript. All authors read and approved the final manuscript.
